# Systematic review and meta-analysis of the efficacy and safety of stem cell treatment of anal fistulas

**DOI:** 10.1007/s10151-025-03138-y

**Published:** 2025-04-09

**Authors:** S. H. Emile, J. Dourado, P. Rogers, A. Wignakumar, N. Horesh, Z. Garoufalia, S. D. Wexner

**Affiliations:** 1 Ellen Leifer Shulman and Steven Shulman Digestive Disease Center, Cleveland Clinic Florida, 2950 Cleveland Clinic Blvd., Weston, FL 33331 USA; 2 Colorectal Surgery Unit, General Surgery Department, Mansoura University Hospitals, Mansoura, Egypt; 3 Department of Surgery and Transplantation, Sheba Medical Center, Ramat-Gan, Israel

**Keywords:** Stem cells, Anal fistulas, Healing, Adverse effects, Systematic review

## Abstract

**Background:**

Since anal fistulas can be challenging to treat; numerous innovative treatments have been proposed, including stem cell therapy. This systematic review aimed to assess pooled rates of fistula healing and adverse events associated with stem cell treatment.

**Methods:**

In this PRISMA-compliant systematic review we searched PubMed and Scopus for observational and randomized studies reporting outcomes of stem cell treatment for anal fistulas. The main outcome measures were successful healing and adverse effects of stem cell therapy.

**Results:**

In total, 43 studies incorporating 1160 patients (53.6% male) were included. Underlying fistula etiologies were Crohn’s disease (30 studies) and cryptoglandular disease (12 studies). The main origin of stem cells was from adipose tissue (34 studies) or bone marrow (6 studies). The median follow-up duration was 12 months. The combined overall pooled healing rate was 58.1% (95% confidence interval (CI) 51.5–64.7%). The pooled healing rate for Crohn’s fistulas was 60.4% (95% CI 54.7–66.2%) with adipose-derived stem cells and 63.6% (95% CI 49.4–77.7%) with bone-marrow-derived cells. The pooled healing rate for cryptoglandular fistulas was 53.8% (95% CI 35.5–72.2%) with adipose-derived stem cells. The pooled complication rate was 37.3% (95% CI 27.1–47.5%). Stem cells were associated with higher odds of anal fistula healing (odds ratio (OR): 1.81, *p* = 0.003) and similar odds of complications (OR: 1, *p* = 0.986) compared with controls.

**Conclusions:**

Stem cell treatment of anal fistulas was associated with promising results. The healing rate in Crohn’s anal fistulas was higher than in cryptoglandular fistulas. Bone-marrow-derived stem cells were associated with marginally better outcomes than were adipose-derived cells. This finding suggests that the autoimmune inflammatory etiology of Crohn’s disease may respond better to autologous myoblasts than does the infectious etiology of cryptoglandular fistulas.

**Supplementary Information:**

The online version contains supplementary material available at 10.1007/s10151-025-03138-y.

## Introduction

Anal fistulas are among the most common anorectal conditions, with an overall prevalence of 18.37 per 100,000 individuals [1]. Although fistulotomy is considered the standard of care for superficial fistulas [2], the management of more complex anal fistulas is more contentious. Complex anal fistulas (CAF) comprise fistulas involving > 30% of the external anal sphincter, and/or suprasphincteric, extra-sphincteric, or horseshoe fistulas [3]. In these scenarios, fistulotomy or fistulectomy may result in a compromise in continence owing to the division of the anal sphincter muscles. The main challenge in the treatment of CAF is the achievement of a fine balance between the need to eradicate the fistula pathology and associated sepsis, while preserving the anal sphincter muscles and continence [4]. To address this objective, several sphincter-preserving procedures have been described, including the placement of setons, ligation of intersphincteric fistula tract (LIFT), endorectal advancement flap, laser therapy, and video-assisted anal fistula treatment [5–8].

Despite the presence of a multitude of sphincter-sparing procedures for CAF, the efficacy of these procedures is limited with no panacea. Moreover, some of these “sphincter-saving” procedures are followed by varying degrees of fecal incontinence [6, 9]. A novel and extraordinary treatment proposed for CAF, particularly those associated with Crohn’s disease, is the use of stem cells. A study [10] suggested that several factors contribute to the pathophysiology of Crohn’s anal fistulas, including high-pressure zone, dead space, sepsis, and by-products of bacterial colonization. The use of stem cells in CAF was based on the theory that the antiinflammatory mechanisms associated with stem cells may help remove the proinflammatory environment that factors into fistula persistence [9].

Early studies on the use of adipose-derived stem cells for the treatment of cryptoglandular and Crohn’s anal fistulas reported promising results, with fistula healing in 71% of patients who were treated with stem cells as compared with 16% in the control group. Healing rates were similar among cryptoglandular or Crohn’s fistulas [11]. A systematic review [12] found that mesenchymal stem cell therapy conferred a higher healing rate than the control group with an odd ratio of 1.95; however, this review only included Crohn’s associated anal fistulas. We hypothesized that the use of stem cells may be an effective and safe treatment of anal fistulas; however, the ultimate efficacy of stem cell therapy may vary according to the type of anal fistula, the origin of stem cells, and differences in the application method, among other factors. Therefore, we conducted the present systematic review to assess the pooled rates of fistula healing and adverse events associated with stem cell treatment of anal fistulas.

## Methods

### Registration and reporting

This systematic review has been reported in adherence with the guideline of Preferred Reporting Items for Systematic Reviews and Meta-Analyses (PRISMA 2020) [13], and the PRISMA checklist is attached in the Supplementary File. We prospectively registered the review protocol in the International Prospective Register of Systematic Reviews “PROSPERO” (CRD42023478666). The original search was conducted in October 2023, as per the registered protocol, and was subsequently updated in December 2024.

### Literature search

A systematic literature search was independently undertaken by two authors (S.E. and P.R.). The authors searched PubMed and Scopus from the inception of each database through December 2024 for published and ahead-of-publication studies on the efficacy of stem cells in the treatment of anal fistulas. To maximize the sensitivity of the search process, the PubMed function “related articles” was activated, and the references section of the studies retrieved was screened to look for further eligible articles, using the snowball search strategy. The clinical trials registry (clinicaltrials.gov) was searched for ongoing clinical trials on stem cell treatment of anal fistulas.

Keywords used in the search process included “stem cells,” “Mesenchymal,” “Adipose-derived,” “Bone marrow,” “Anal fistula,” “Perianal fistula,” “Anorectal fistula,” “outcome,” “Healing,” and “Efficacy”. In addition, we used the following medical subject headings (MeSH) terms: (Rectal fistula), (Stem cells), and (Healing). The following syntax combination was used for the literature search: (Mesenchymal OR Adipose-derived OR Bone marrow) AND (Stem cell) AND (Anal fistula OR perianal fistula OR Anorectal fistula) AND (Outcome OR Healing OR Efficacy OR Failure).

After the exclusion of duplicate reports and conference abstracts without sufficient information, the remaining studies were screened by title and abstract, excluding irrelevant studies, then the full-text versions of the retained articles were independently reviewed by one of three authors (J.D., P.R., or A.W.) to check for eligibility. The first author (S.E.) ultimately reviewed the results of data extraction. The process of article selection and screening was supervised by the senior author (S.D.W).

### Study selection

Study designs deemed eligible for inclusion were observational studies (pilot, cohort, and case–control studies) and randomized clinical trials (RCTs). The following PICO criteria were used to guide the inclusion of the studies:

- P (*Patients*): patients with anal fistulas, whether cryptoglandular or associated with Crohn’s disease.
- I (*Intervention*): stem cell therapy.
- C (*Comparator*): normal saline, fibrin, other surgical procedure, placebo, or no comparator.
- O (*Outcome*): healing and complications.

We excluded animal studies, case reports, case series including less than five patients, editorials, previous reviews and meta-analyses, and studies that did not report healing after stem cell treatment. There were no restrictions imposed on the language of the studies or follow-up duration. If two studies included overlapping data, only the most recent or larger study reporting the outcomes of this review was selected for inclusion.

### Assessment of study quality and risk of bias

Three authors (J.D., P.R., and A.W.) independently assessed the risk of bias in the studies using the ROBINS-1 tool [14] for observational studies and the risk of bias-2 (ROB-2) tool [15] for RCTs. Conflicts in assessments were resolved by discussion and mutual agreement. The publication bias was assessed by visual inspection of a funnel plot of the standard error of the rates of each outcome against the rates of the outcome. The absence of publication bias was indicated by the symmetry of the funnel plot and the presence of 95% of the studies near the straight vertical line. The certainty of the evidence of each outcome was graded using the Grading of Recommendations Assessment, Development and Evaluation (GRADE) approach [16] as very low, low, moderate, and high.

### Data collected

Three authors (J.D., P.R., and A.W.) extracted the following data points from the studies into an Excel sheet template:

- Authors, country, and design of the study.
- Number and demographics of patients treated with stem cells, including age, sex, body mass index (BMI), and smoking status.
- Fistula-related data, including type and origin of anal fistula, duration of disease, previous fistula treatments, and rectal involvement by Crohn’s disease.
- Stem cell treatment: type, origin, and dose of stem cells, method of application, and any adjunct methods used.
- Healing rate.
- Number and type of complications of stem cell therapy.
- Follow-up duration.

### Outcome measures

The primary outcome was the efficacy of stem cell therapy, assessed by healing at the end of follow-up. Healing was defined as the clinical complete healing of anal fistulas with the absence of discharge and epithelization of the external opening. The secondary outcomes included complications and adverse events after stem cell treatment.

### Statistical analysis

The open-source, cross-platform software for advanced meta-analysis “openMeta [Analyst]™” version 12.11.14. was used to conduct proportional meta-analyses. The weighted pooled rates of healing and complications along with their corresponding 95% confidence intervals (CI) were calculated using the DerSimonian and Laird random-effect model. Heterogeneity was assessed using the inconsistency (*I*^2^) statistics (low if *I*^2^ < 25%, moderate if *I*^2^ = 25–75%, and high if *I*^2^ > 75%). A random-effects pair-wise meta-analysis was conducted, including only RCTs to estimate the odds ratio (OR) of healing and complications in the stem cell group compared with the control group. A random-effect meta-regression analysis was undertaken to determine the factors significantly associated with the healing of anal fistulas. The results of meta-regression were reported as slope coefficient (SE) and *p* value. Owing to the low statistical power of meta-regression analyses, a *p* value < 0.1 was considered statistically significant.

## Results

### Characteristics of patients and studies

**Fig. 1:**
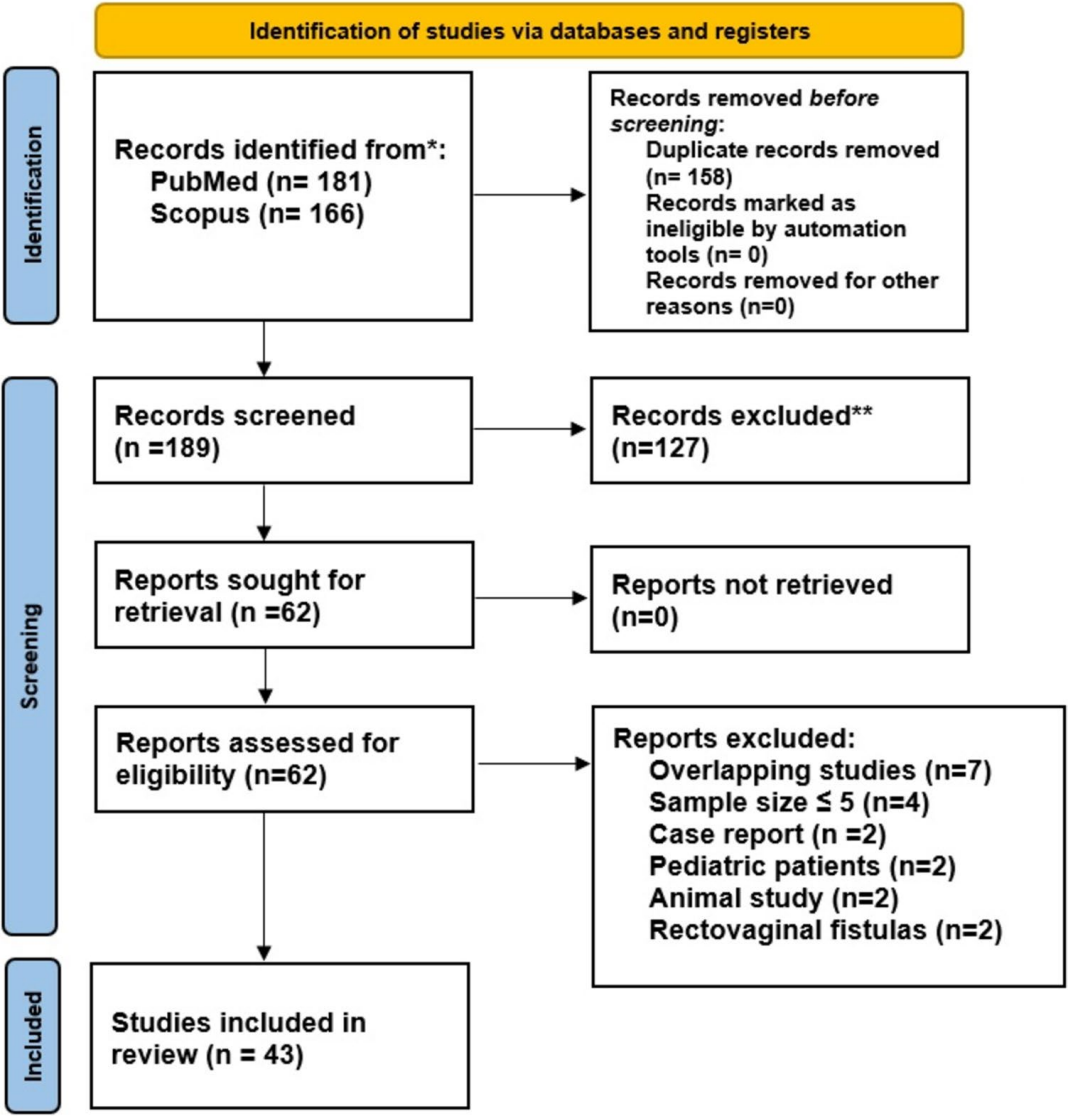
PRISMA flow chart for study inclusion

After screening the records of 189 studies, 43 studies [17–59] were included in this meta-analysis (Fig. 1). The studies were published between 2011 and 2024 and conducted in Europe (*n* = 23), Asia (*n* = 11), the USA (*n* = 5), Mexico (*n* = 1), and Australia (*n* = 1). Two studies were international multicentric. In addition, 10 studies were RCTs, and 33 were observational cohort or pilot studies.

The studies included 1160 patients (53.6% male) with a median age of 37.6 (range 24.4–50.4) years and a median BMI of 24.9 (range 21.7–32) kg/m^2^. Previous fistula treatment was administered to 833 (71.8%) patients. Anal fistulas were associated with Crohn’s disease in 30 studies and were cryptoglandular in 12 studies, whereas 1 study included both types of anal fistulas. The median duration of the disease was 91.8 (range 10.3–228) months. The characteristics of the studies are shown in Table 1. Searching the clinical trial registry revealed nine ongoing trials (NCT05974280, NCT00115466, NCT04750499, NCT05177003, NCT05039411, NCT05075811, NCT05402748, NCT04519684, and NCT05709717).

**Table 1:** Characteristics of the studies included

Study	Country	Design	Control group	Number	Male	Age	Previous treatment	Type of anal fistula	Follow-up (months)
Guillaumes et al., 2024	Spain	Prospective cohort	None	9	6	42	9	Cryptoglandular	18
Pronk et al., 2024	The Netherlands	Retrospective cohort	None	30	15	34.5	30	Crohn’s disease	16.5
Swaroop et al., 2024	India	Phase I/II clinical trial	None	10	8	27.4	5	Crohn’s disease	26
Herreros et al., 2024	Spain	Observational retrospective cohort, multicenter	None	73	36	42.5	66	Crohn’s disease	6
Park et al. 2024	South Korea	Observational retrospective	Anti-TNF	65	29	26	65	Crohn’s disease	65.9
Lightner et al., 2024	USA	Phase IB/IIA RCT	Placebo	15	NR	38.3	15	Crohn’s disease	12
White et al., 2024	Israel	Prospective multicentric	None	33	14	35.1	11	Crohn’s disease	14
Keung et al., 2023	Australia	Phase I trial	None	10	3	37	7	Crohn’s disease	13
Fathallah et al., 2023	France	Retrospective, multicenter	None	43	22	37	43	Crohn’s disease	12.7
Wei et al., 2023	China	Prospective single-arm pilot	None	10	8	34.3	17	Crohn’s disease	6
Dawoud et al., 2023	Austria	Prospective multicenter cohort	None	14	3	32	14	Crohn’s disease	23
Arkenbosch et al., 2023	The Netherlands	Prospective pilot	None	25	11	35	25	Crohn’s disease	12
Dalby et al., 2023	Denmark	Prospective cohort	None	77	28	46.9	23	Cryptoglandular	6
Reenaers et al., 2023	Belgium	Prospective monocentric cohort	None	16	8	49	3	Crohn’s disease	12
Pak et al., 2023	Iran	Phase I trial	None	11	8	43.27	11	Cryptoglandular	6
Lightner et al., 2023	USA	Phase IB/IIA RCT	Normal saline (5)	18	7	34	18	Crohn’s disease	6
Dozois et al., 2023	USA	Phase I trial	None	20	8	36	20	Crohn’s disease	12
Furukawa et al., 2023	Japan	Phase III single-arm trial	None	22	14	36.4	NR	Crohn’s disease	13
Guillo et al., 2022	France	Single-arm phase I trial	None	10	6	37.3	10	Crohn’s disease	36
Garcia-Olmo et al., 2022	Multicenter	Phase 3 double-blind RCT	Normal saline (15)	25	14	38.6	21	Crohn’s disease	26
Sørensen et al., 2022	Denmark	Prospective single-center pilot	None	12	3	33	9	Crohn’s disease	12
Tencerova et al., 2021	Denmark	Prospective cohort	None	27	9	45	9	Cryptoglandular	6
Schwandner et al., 2021	Germany	Retrospective cohort	None	12	6	42.5	12	Crohn’s disease	14.3
Cabalzar-Wondberg et al., 2021	Switzerland	Prospective case series	None	11	8	38.3	11	Crohn’s disease	10.4
Ascanelli et al., 2021	Italy	RCT	No intervention (58)	58	37	50.41	24	Cryptoglandular	6
Maciel Gutiérrez et al., 2021	Mexico	Phase I trial	None	20	13	39.6	NR	Cryptoglandular	6
Zhang et al., 2020	China	Prospective case–control	Advancement flap (13)	11	10	35.7	NR	Cryptoglandular	3
Laureti et al., 2020	Italy	Prospective pilot	None	15	7	40.1	15	Crohn’s disease	6
Zhou et al., 2020	China	Open label RCT	Incision-thread-drawing procedure (11)	11	11	24.4	NR	Crohn’s disease	12
Garcia-Arranz et al., 2020	Spain	Multicenter single-blind RCT	Fibrin glue (21)	23	16	50.1	NR	Cryptoglandular	24
Barnhoorn et al., 2020	The Netherlands	Placebo controlled RCT	Placebo (6)	13	8	43	NR	Crohn’s disease	48
Topal et al., 2019	Turkey	Prospective cohort	None	10	8	47	5	Cryptoglandular	9
Dige et al., 2019	Denmark	Prospective single arm	None	21	6	NR	21	Crohn’s disease	6
Dozois et al., 2019	USA	Phase I prospective trial	None	15	7	39.8	12	Cryptoglandular	6
Panes et al., 2018	Multicenter	Phase III, double-blind RCT	Placebo (105)	107	60	39	107	Crohn’s disease	13
Choi et al., 2017	Korea	Multicenter phase II trial	None	15	15	37.9	12	Cryptoglandular	6
Dietz et al., 2017	USA	Phase I trial	None	12	6	32	12	Crohn’s disease	6
Cho et al., 2015	Korea	Multicenter phase II trial	None	41	28	26.2	26	Crohn’s disease	24
Park et al. 2015	Korea	Prospective pilot	None	6	4	32.2	4	Crohn’s disease	8
Guadalajara et al., 2012	Spain	Phase II, multicenter RCT	Fibrin (25)	24	10	42.6	17	Crohn’s disease and cryptoglandular	38
de la Portilla et al., 2012	Spain	Multicenter, Phase I/IIa trial	None	24	11	36	NR	Crohn’s disease	6
Herreros et al., 2012	Spain	Phase III multicenter RCT	Fibrin and saline (59)	124	83	48.5	112	Cryptoglandular	6
Ciccocioppo et al., 2011	Italy	Prospective cohort	None	12	8	32	12	Crohn’s disease	12

*NR* not reported, *RTC* randomized control trial

### Stem cell therapy

Mesenchymal stem cells were used in 29 studies, whereas the remaining studies used adipose-derived stem cells (6 studies), stromal vascular fraction (3 studies), freshly collected autologous adipose tissue (4 studies), or epithelial cells (1 study). The source of stem cells was from the adipose tissue (34 studies) followed by bone marrow (6 studies), placenta/amniotic membrane (2 studies), and the umbilical cord (1 study). Autologous stem cells were used in 24 studies and allogenic stem cells in 19 studies. Most studies injected stem cells around the internal opening and along the fistula tract, whereas some studies injected stem cells in fibrin through the fistula tract and others used a stem-cells-coated plug. Closure of the internal fistula orifice was undertaken in 34 studies. A summary of the technical details of stem cell application in the studies is presented in Table 2.

**Table 2:** Technical aspects of stem cell therapy of anal fistulas

Study	Type	Origin	Source	Method of application	Dose	Adjuncts
Guillaumes et al., 2024	Autologous adipose tissue	Adipose tissue	Autologous	Around the IO and mucosal flap	10–15 mL	IO closure, curetting the fistula tract
Pronk et al., 2024	Mesenchymal	Adipose tissue	Allogenic	Around the IO and along the fistula tract	24 mL	IO closure, curetting the fistula tract
Swaroop et al., 2024	Mesenchymal	Bone marrow	Allogenic	Around the IO and along the fistula tract	15 mL, 5 × 10^6^ cells/mL	IO closure, curetting the fistula tract
Herreros et al., 2024	Mesenchymal	Adipose tissue	Allogenic	Around the IO and along the fistula tract	120 million cells	IO closure, curetting the fistula tract
Park et al. 2024	Autologous adipose tissue	Adipose tissue	Autologous	Around the IO and along the fistula tract	6 × 10^7^ cells per cm	IO closure, curetting the fistula tract
Lightner et al., 2024	Mesenchymal	Bone marrow	Allogenic	Around the IO and along the fistula tract	7.5 mL, 75 million cells	IO closure, curetting the fistula tract
White et al., 2024	Mesenchymal	Adipose tissue	Allogenic	Around the IO and along the fistula tract	120 million cells	IO closure, curetting the fistula tract
Keung et al., 2023	Epithelial	Amniotic membrane	Allogenic	Around the IO and along the fistula tract	40 million cells per 4.5 ml	IO closure, curetting the fistula tract
Fathallah et al., 2023	Mesenchymal	Adipose tissue	Allogenic	Around the IO and along the fistula tract	120 million cells	IO closure
Wei et al., 2023	Mesenchymal	Umbilical cord	Allogenic	Half along the tract, half around the IO	120 million stem cells suspended in 24 mL	IO closure
Dawoud et al., 2023	Mesenchymal	Adipose tissue	Allogenic	Half along the tract, half around the IO	1 mL SVF	IO closure
Arkenbosch et al., 2023	Stromal vascular fraction	Adipose tissue	Autologous	Injected around the IO and into all quadrants of the fistula wall along the fistula tract	NA	PRP + IO closure
Dalby et al., 2023	Autologous adipose tissue	Adipose tissue	Autologous	Around the IO and along the fistula tract	3 × 10^7^ MSCs	IO closure, curetting the fistula tract
Reenaers et al., 2023	Mesenchymal	Bone marrow	Autologous	Half along the tract, half around the IO	50 μg/mL	None
Pak et al., 2023	Mesenchymal	Human placenta	Allogenic	Injected along the fistula tract near the internal orifice weekly	1–2 doses 75 million in 7.5 mL	None
Lightner et al., 2023	Mesenchymal	Bone marrow	Allogenic	Two thirds injected along the tract, and one third injected under the IO	6 tubes of the MSC-MATRIX	IO closure
Dozois et al., 2023	Mesenchymal	Adipose tissue	Autologous	The disk of the MSC-MATRIX was sutured to the rectal wall at the IO	24 mL containing 120 × 10^6^ cells	None
Furukawa et al., 2023	Mesenchymal	Adipose tissue	Allogenic	Half injected along the tract, half near the IO	22.8 × 10^6^ cells	IO closure
Guillo et al., 2022	Stromal vascular fraction	Adipose tissue	Autologous	Injected into the wall of the fistula and surrounding tissues	5 × 10^6^ cells/mL; 120 × 10^6^ cells	IO closure
Garcia-Olmo et al., 2022	Mesenchymal	Adipose tissue	Autologous	Injected around the IO	30–50 mL	IO closure
Sørensen et al., 2022	Stromal vascular fraction	Adipose tissue	Autologous	Injected around the entire length of fistula tract	NA	IO closure
Tencerova et al., 2021	Mesenchymal	Adipose tissue	Autologous	Injected around and into the fistula tract	120 million stem cells	IO closure
Schwandner et al., 2021	Mesenchymal	Adipose tissue	Allogenic	Half along the tract, half around the IO	16 mL	IO closure
Cabalzar-Wondberg et al., 2021	Mesenchymal	Adipose tissue	Allogenic	Half along the tract, half around the IO	40 × 10^6^ ASCs	IO closure
Ascanelli et al., 2021	Adipose-derived stem cells	Adipose tissue	Autologous	4 cc injected around the IO, 2 cc along the tract or inside the perianal wound	5 × 10^6^ cells/mL	IO closure, fistulectomy, flap, VAAFT
Maciel Gutiérrez et al., 2021	Mesenchymal	Adipose tissue	Allogenic	Injected around the IO and into the wall of the tract + injected inside the tract	20 cc	IO closure
Zhang et al., 2020	Adipose-derived stem cells	Adipose tissue	Autologous	Injected around the IO and tract then infused through the external opening	5 × 10^6^ cells/mL	IO and EO closure
Laureti et al., 2020	Microfragmented adipose tissue	Adipose tissue	Autologous	Injected around the IO and along fistula tract	100 million ASCs plus fibrin	Cone-like fistulectomy
Zhou et al., 2020	Adipose-derived stem cells	Adipose tissue	Autologous	Into the IO and around the fistula wall	1 × 10^7^, 3 × 10^7^, or 9 × 10^7^	IO closure
Garcia-Arranz et al., 2020	Mesenchymal	Adipose tissue	Autologous	Half of dose injected around IO and half through the external opening	10 mL	Surgery protocol
Barnhoorn et al., 2020	Mesenchymal	Bone marrow	Allogenic	Half of the dose injected in the wall along the tract, half around the IO	18–104 mL	None
Topal et al., 2019	Adipose-derived stem cells	Adipose tissue	Autologous	Around the IO and inside the fistula tract wall	NA	IO closure
Dige et al., 2019	Autologous adipose tissue	Adipose tissue	Autologous	Injected around the IO and around the tract	120 million Cx601 cells	The fistula tract was cut transversely
Dozois et al., 2019	Mesenchymal	Adipose tissue	Autologous	MSC-MATRIX plug was passed through the tract from the IO through the external opening	1–2 × 10^7^ cells/mL	IO sealed by the cap of the plug
Panes et al., 2018	Mesenchymal	Adipose tissue	Allogenic	Half injected to IO and half through the external opening into the fistula walls	20 × 10^6^ cells per plug	IO closure
Choi et al., 2017	Adipose-derived stem cells	Adipose tissue	Autologous	Injected evenly into the submucosal layer around the IO and inside of the tract + fibrin glue injection	3 × 10^7^ cells per centimeter length	IO closure and fibrin glue
Dietz et al., 2017	Mesenchymal	Adipose tissue	Autologous	MSC-MATRIX plug	1 × 10^7^ cells/mL (first group), 3 × 10^7^ cells/ml (2nd group)	None
Cho et al., 2015	Mesenchymal	Adipose tissue	Autologous	Injected into the submucosa around IO and in the fistula tract wall; open fistula tract was filled with a mixture of ASCs and fibrin glue	Fibrin glue plus 2 × 10^7^ ASCs	IO closure and fibrin glue
Park et al. 2015	Mesenchymal	Adipose tissue	Allogenic	Injected to submucosa of the IO, then fistula track filled with a mixture of ASCs and fibrin glue	2 × 10^7^ cells first dose, 2 × 10^7^ cells second dose	IO closure and fibrin glue
Guadalajara et al., 2012	Mesenchymal	Adipose tissue	Autologous	Half injected in the intersphincteric tract near the IO and half in the tract wall	Group 1, 2 × 10^7^ million ASCs; group 2, 2 × 10^7^ ASCs plus fibrin glue	IO closure and fibrin glue
de la Portilla et al., 2012	Mesenchymal	Adipose tissue	Allogenic	Half injected in the intersphincteric tracts and half in the tract walls	20 × 10^6^ cells every 4 weeks	IO closure
Herreros et al., 2012	Mesenchymal	Adipose tissue	Autologous	Half injected around the IO and half to walls of tract with or without injection of fibrin in the tract	120 million cells	IO closure and fibrin glue
Ciccocioppo et al., 2011	Mesenchymal	Bone marrow	Autologous	Injected into the lumen and the wall of the tracks	120 million stem cells suspended in 24 mL	None

*EO* external opening, *IO* internal opening, *NA* not available, *SVF* stromal vascular fraction, *MSC* mesenchymal stem cells, *VAAFT* video-assisted anal fistula treatment, *ASC* adult stem cells

### Efficacy

**Fig. 2:**
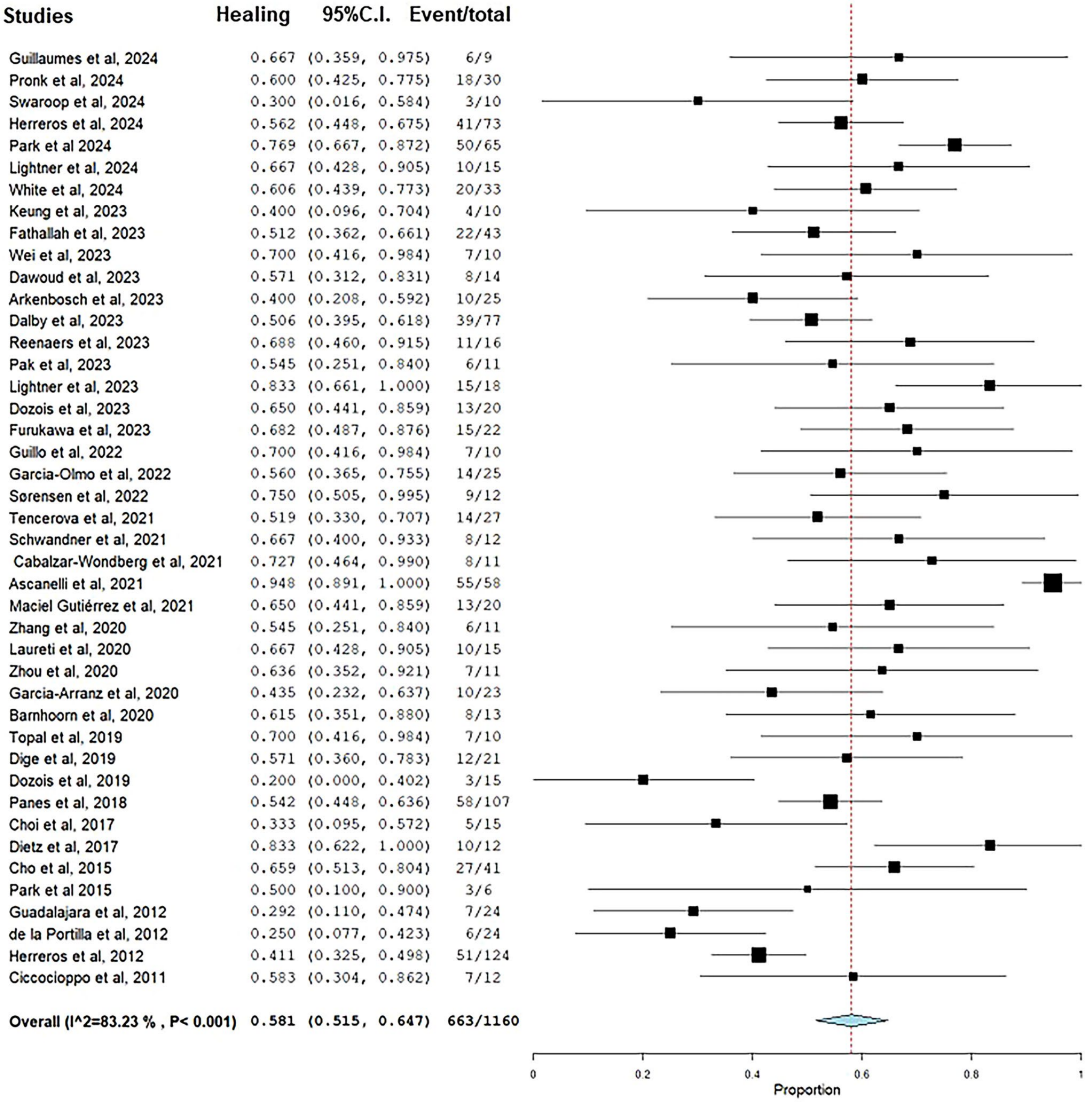
Pooled rate of healing after stem cell treatment of anal fistulas

The median follow-up duration was 12 (range, 3–66) months. Complete healing of anal fistulas was achieved at the end of follow-up in 663 (57.1%) patients. The pooled healing rate was 58.1% (95% CI 51.5–64.7%) with a significant statistical heterogeneity (*I*^2^ = 83.2%) (Fig. 2). Healing was defined on the basis of clinical and radiologic examination by magnetic resonance imaging (MRI) in 24 studies, whereas 19 studies used a clinical definition of healing. The detailed definition of complete healing of anal fistulas reported in the studies is shown in Appendix Table 1.

#### Sensitivity analyses

**Fig. 3:**
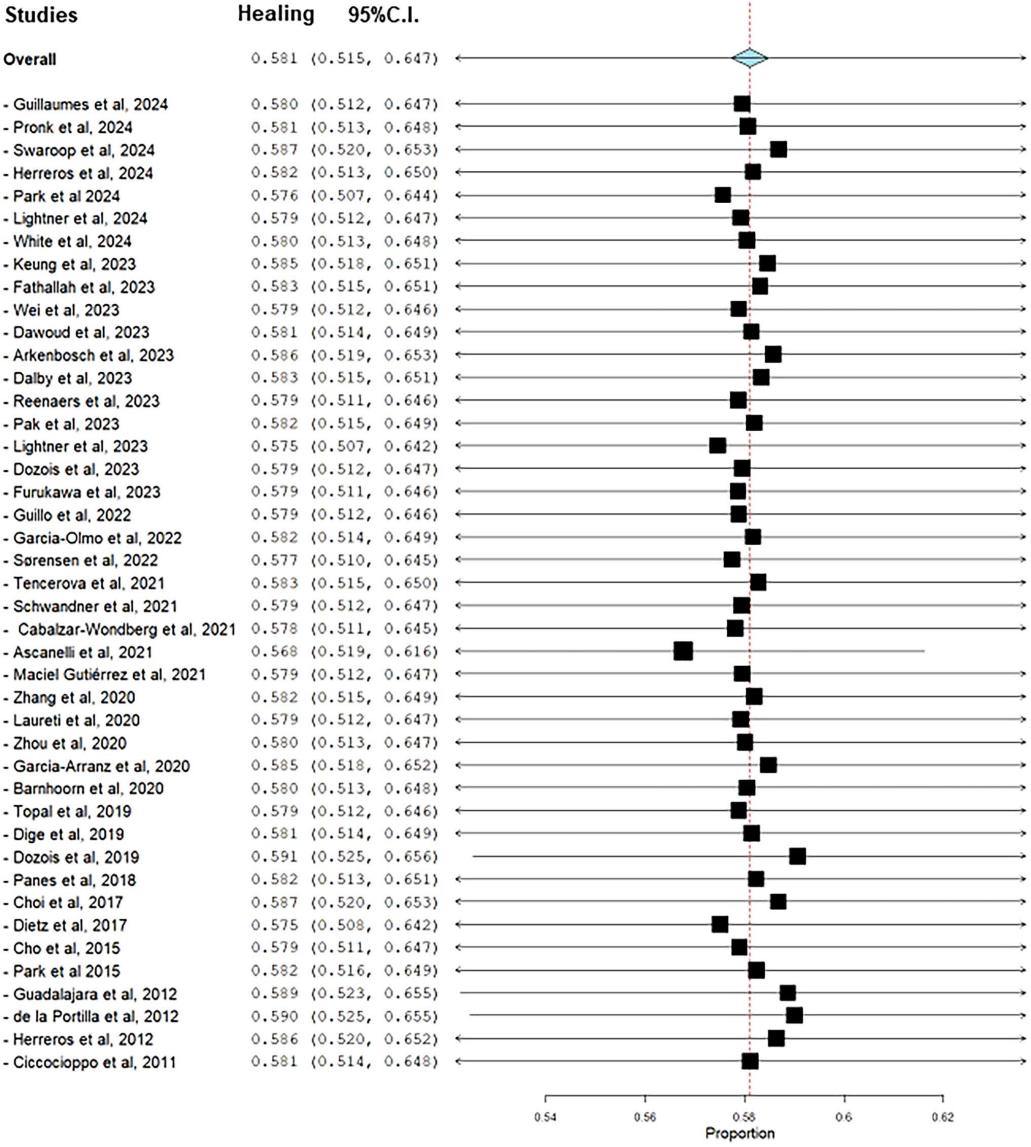
Leave-one-out meta-analysis of healing after stem cell treatment of anal fistulas

A leave-one-out meta-analysis showed that the exclusion of each study did not reveal a large study effect as the original effect estimate did not change by > 10% when each study was omitted from the pooled analysis (Fig. 3).

Analysis of eight studies that included patients with a mean BMI > 25 kg/m^2^ revealed a pooled healing rate of 62.5% (95% CI 42.6–82.5%). Studies that included only patients with Crohn’s fistulas (*n* = 30) had a pooled healing rate of 60.9% (95% CI 55.8–66%) compared with 53.9% (95% CI 36.5–71.3%) for the studies that only included cryptoglandular fistulas (*n* = 12). Adipose tissue-derived stem cells (34 studies) yielded a pooled healing rate of 57.6% (95% CI 50–65.2) compared with 63.6% (95% CI 49.4–77.7) for bone marrow-derived stem cells (6 studies) and 47.5% (95% CI 26.4–68.6) for amniotic membrane/placenta-derived stem cells (2 studies). Autologous stem cells (24 studies) conferred a pooled healing rate of 58.4% (95% CI 48.3–68.5%) compared with 57.7% (95% CI 51.4–64%) for allogeneic stem cells (19 studies).

Closure of the internal fistula orifice was performed in 32 studies and was associated with a pooled healing rate of 57.1% (95% CI 49.3–64.9%). Studies with a follow-up of ≥ 12 months (*n* = 24) had a pooled healing rate of 58.4% (95% CI 52.9–63.9%) compared with 58% (95% CI 45.6–70.4%) for studies with follow-up < 12 months (19 studies). Studies that used both clinical and radiologic definitions of healing (*n* = 24) had a pooled healing rate of 56.5% (95% CI 50.1–62.9%) compared with 59.6% (95% CI 48.7–70.4%) for the studies that used only a clinical definition of healing (*n* = 19). Table 3 summarizes the main sensitivity analyses of pooled healing after stem cell therapy.

**Table 3:** Pooled healing rates in different subgroups

Group	Number of studies	Pooled healing (%)	95% confidence interval
Overall	43	58.1	51.5–64.7
Increased body mass index	8	62.5	42.6–82.5
Crohn’s fistulas	30	60.9	55.8–66
Cryptoglandular fistulas	12	53.9	36.5–71.3
Adipose tissue-derived	34	57.6	50–65.2
Bone marrow-derived	6	63.6	49.4–77.7
Amniotic membrane/placenta-derived	2	47.5	26.4–68.6
Autologous stem cells	24	58.4	48.3–68.5
Allogenic stem cells	19	57.7	51.4–64
Follow-up ≥ 12 months	24	58.4	52.9–63.9
Follow-up < 12 months	19	58	45.6–70.4

**Fig. 4:**
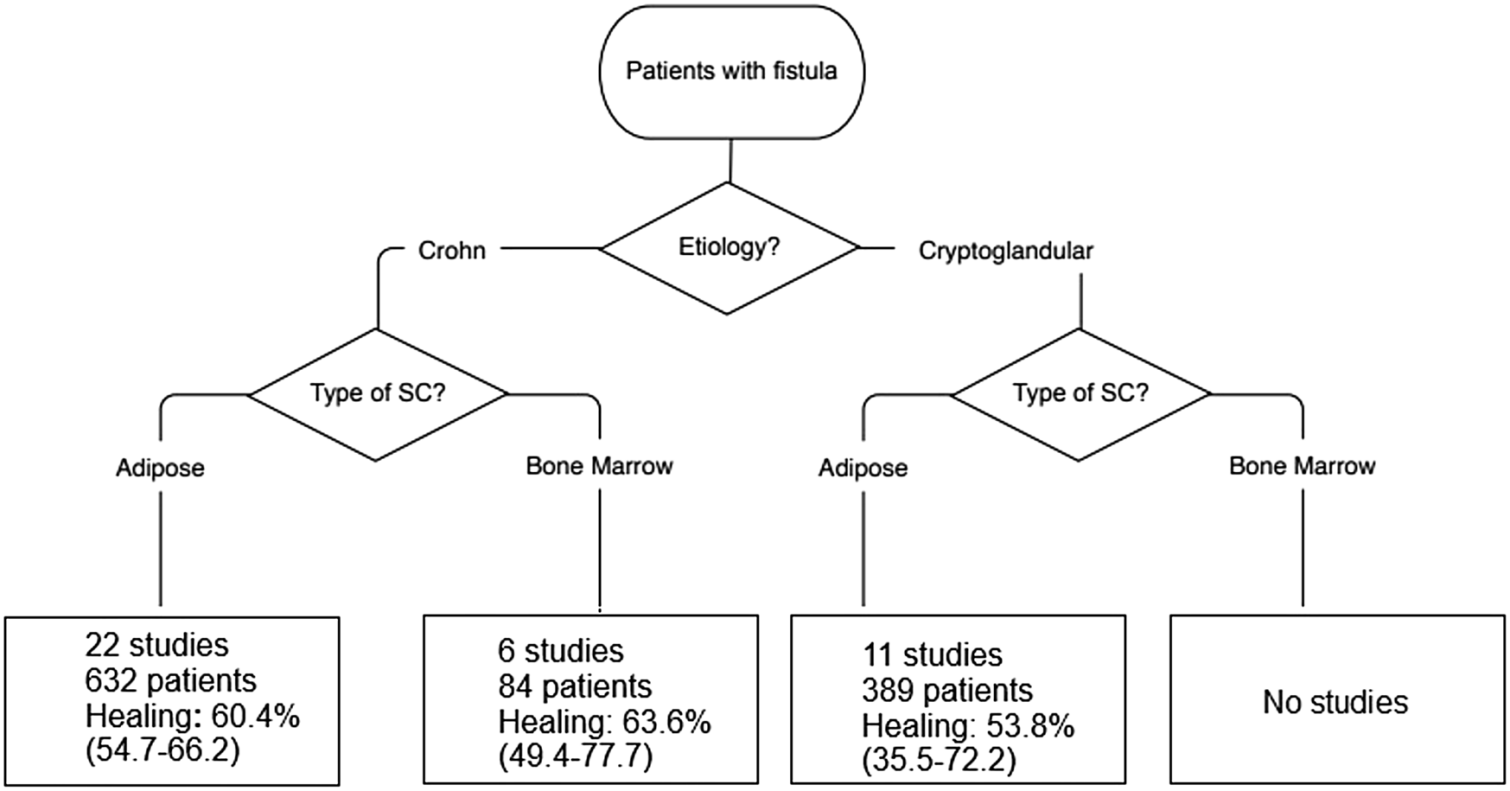
Pooled healing rates after stem cell treatment of anal fistulas in different subgroups

Figure 4 illustrates the pooled healing rates in subgroups of patients according to the fistula type and source of stem cells. The pooled healing rate for Crohn’s fistulas was 60.4% (95% CI 54.7–66.2%) with adipose-derived stem cells (22 studies, 632 patients) and 63.6% (95% CI 49.4–77.7%) with bone-marrow-derived cells (6 studies, 84 patients). The pooled healing rate for cryptoglandular fistulas was 53.8% (95% CI 35.5–72.2%) with adipose-derived stem cells (11 studies, 389 patients).

Meta-regression analyses showed that smoking (standard error (SE)= −0.011, *p* < 0.001) and rectal involvement by Crohn’s disease (SE = 0.008, *p* = 0.054) were significantly associated with poorer healing after stem cell injection. Nonsignificant factors included sex (SE = −0.001, *p* = 0.766), age (SE = − 0.002, *p* = 0.657), BMI (SE = 0.002, *p* = 0.889), previous fistula treatment (SE = −0.001, *p* = 0.524), type of anal fistula (SE = −0.064, *p* = 0.271), disease duration (SE = 0.0001, *p* = 0.489), stem cell origin (SE = 0.001, *p* = 0.984), follow-up duration (SE = 0.001, *p* = 0.683), and complications (SE: −0.001, *p* = 0.416).

### Safety

**Fig. 5:**
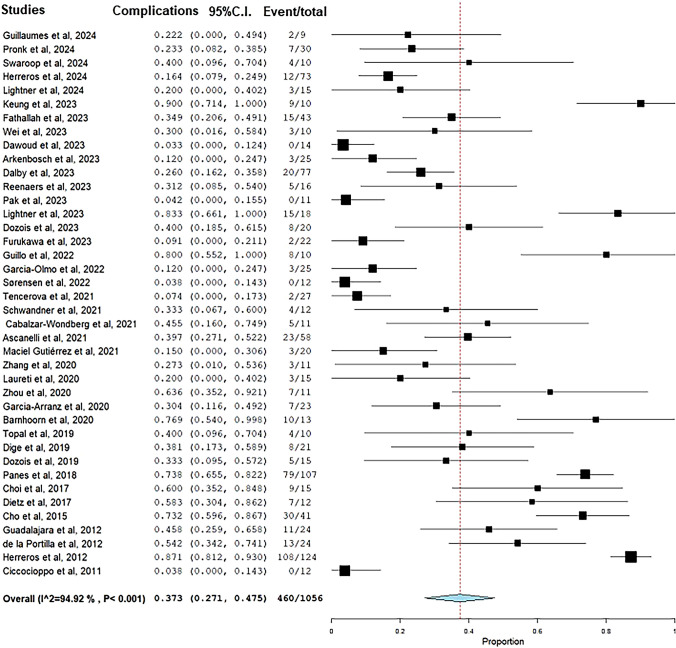
Pooled rate of complications after stem cell treatment of anal fistulas

Complications were recorded in 460 patients with a pooled complication rate of 37.3% (95% CI 27.1–47.5%, *I*^2^ = 95%) (Fig. 5). The pooled rate of abscess was 11.6% (95% CI 8.3–14.9%), and anal pain was 22% (13.2–30.9%). The outcomes of stem cell therapy are detailed in Table 4.

**Table 4:** Outcomes of stem cell treatment of anal fistulas

Study	Healing	Complications	Types of complications
Guillaumes et al., 2024	6	2	Deterioration of continence (1), bleeding (1)
Pronk et al., 2024	18	7	Wound necrosis (1), abscess (6)
Swaroop et al., 2024	3	4	Spinal headache (2), ecchymosis (1), abscess (1)
Herreros et al., 2024	41	12	Abscess (9), cellulitis (1), bleeding (1)
Park et al. 2024	50	NR	NR
Lightner et al., 2024	10	3	Abscess (3)
White et al., 2024	20	NR	Pain, bleeding, fever, abscess
Keung et al., 2023	4	9	Pain (7), abscess (2)
Fathallah et al., 2023	22	15	Abscess (9), pain (6)
Wei et al., 2023	7	3	Anal pain (2), elevated liver enzymes (1)
Dawoud et al., 2023	8	0	NA
Arkenbosch et al., 2023	10	3	Bleeding (2), wound infection (1)
Dalby et al., 2023	39	20	Abscess (2), bleeding (2), infection (6), infected hematoma (1), anal pain (6), urine retention (3)
Reenaers et al., 2023	11	5	Mild anal pain (3), mild bleeding (1), increased discharge (1)
Pak et al., 2023	6	0	NA
Lightner et al., 2023	15	15	Anal pain (13), abscess (2)
Dozois et al., 2023	13	8	Abscess (4), new tract (2), anal pain (1), non-healing wound (1)
Furukawa et al., 2023	15	2	Worse Crohn’s and diarrhea (1), increase in blood bilirubin (1)
Guillo et al., 2022	7	8	Anal pain (4), abscess (3), new fistula tract (1)
Garcia-Olmo et al., 2022	14	3	Fistula (1), Abscess (1), Increased discharge (1)
Sørensen et al., 2022	9	0	NA
Tencerova et al., 2021	14	2	Abscess (2), anal pain
Schwandner et al., 2021	8	4	Abscess (4)
Cabalzar-Wondberg et al., 2021	8	5	Abscess (4), cytomegalovirus viraemia (1)
Ascanelli et al., 2021	55	23	Abscess (1), hemorrhoids (11), abdominal pain (11)
Maciel Gutiérrez et al., 2021	13	3	Abscess (3)
Zhang et al., 2020	6	3	Anal pain (3)
Laureti et al., 2020	10	3	Bleeding (1), subcutaneous hematoma (2)
Zhou et al., 2020	7	7	Anal pain (7), abscess (3), pyrexia (3)
Garcia-Arranz et al., 2020	10	7	Abscess, back pain, urticaria, renal colic
Barnhoorn et al., 2020	8	10	Abscess (4), infection (4), Crohn’s activity (2)
Topal et al., 2019	7	4	Abscess (2), Bruising at the liposuction site (2)
Dige et al., 2019	12	8	Abscess (2), anal pain (4), bleeding (1), urine retention (1)
Dozois et al., 2019	3	5	Abdominal wall seroma (1), abscess (1), fall of plug and abscess (1), abscess at 3 months (1), perianal cellulitis (1)
Panes et al., 2018	58	79	Anal pain (15), anal abscess/fistula (34), nasopharyngitis (11), diarrhea (9), abdominal pain (5), pyrexia (6)
Choi et al., 2017	5	9	Anal pain (2), bleeding (2), abscess (2), pyrexia (3)
Dietz et al., 2017	10	7	Debridement of granulation tissue in the fistula tract (1), seroma (2)
Cho et al., 2015	27	30	Anal pain (8), bleeding (3), anal inflammation (3), diarrhea (3), pyrexia (3), abdominal pain (7), disease exacerbation (4)
Park et al. 2015	3	12	Anal pain (5), abscess (1), infection (1), fever (1), abdominal pain (1), diarrhea (1), numbness (1), erythema (1)
Guadalajara et al., 2012	7	11	Abscess (1), perianal sepsis (3)
de la Portilla et al., 2012	6	13	Abscess (4), pyrexia (4), anal fistula infection (1), anal pain (2)
Herreros et al., 2012	51	108	Proctalgia (80), abscess (41), pain (25), abscess (24), pyrexia (17), swelling (12), pruritis (12)
Ciccocioppo et al., 2011	7	0	Not reported

### Pairwise meta-analysis of RCTs

**Fig. 6:**
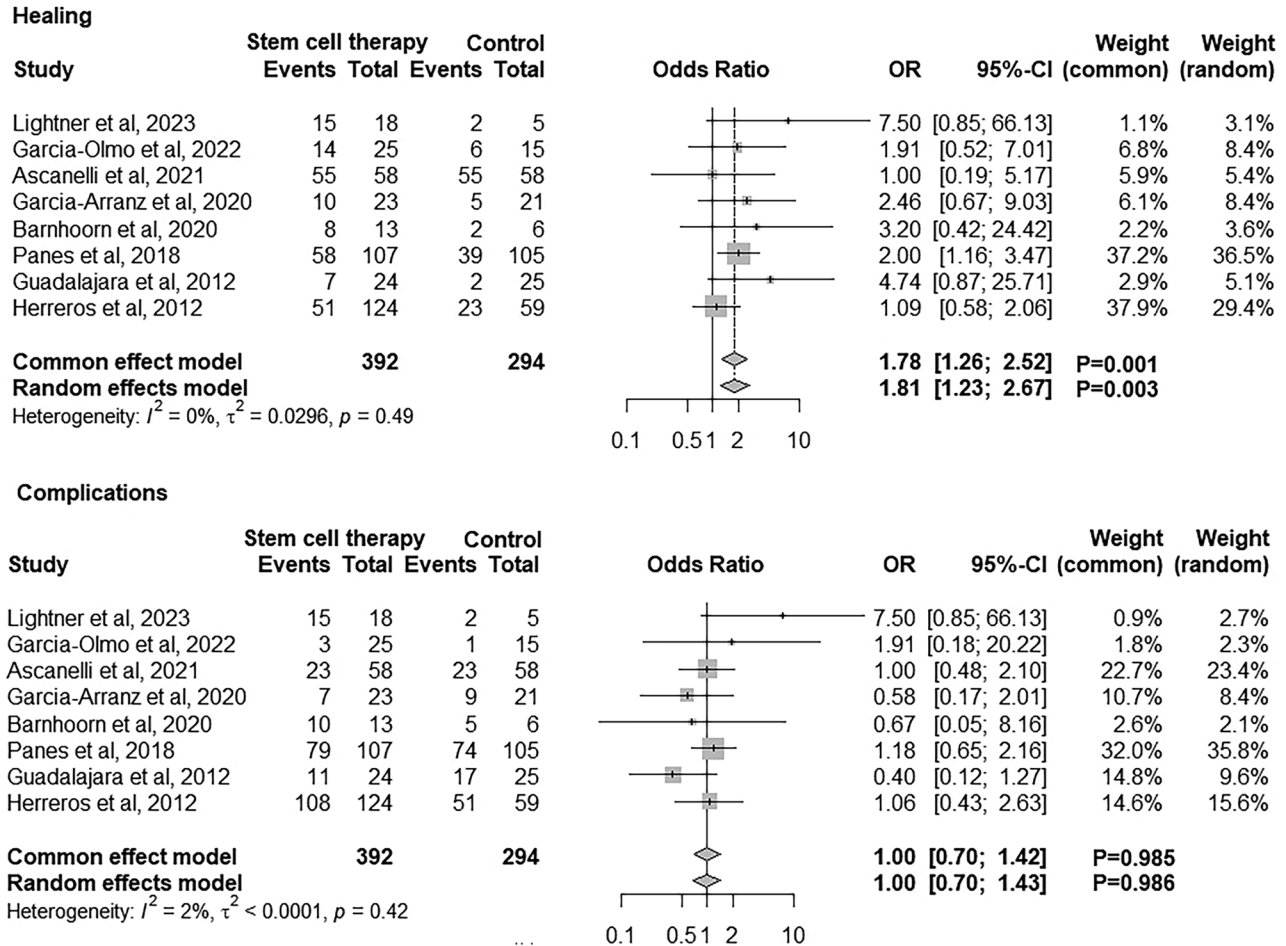
Forest plots illustrating the difference in healing and complications between stem cell treatment and control group

Meta-analysis of eight RCTs that included saline or placebo as a control showed that the use of stem cells was associated with higher odds of healing of anal fistulas (OR: 1.81, 95% CI 1.23; 2.67, *p* = 0.003) and similar odds of complications (OR: 1, 95% CI 0.70; 1.43, *p* = 0.986) compared with the control group (Fig. 6).

### Risk of bias and grade of certainty

According to the ROBINS-1 and RoB-2 tools, 23 studies had a moderate risk of bias, 18 had a high or critical risk of bias, and 2 had a low risk of bias (Appendix Tables 2 and 3; Supplementary Fig. 1). There was no significant publication bias in reporting the main outcomes of the studies (Supplementary Fig. 2). Assessment of certainty of evidence from observational nonrandomized studies showed a very low certainty for healing and complications, whereas assessment of evidence from randomized trials showed a moderate certainty for both outcomes (Appendix Table 4).

There were conflicts of interest reported by the authors of 25 studies. In 15 studies, the authors declared that they had no did not have any conflicts of interest to disclose, while in 3 studies no disclosures were reported (Appendix Table 5).

## Discussion

Treatment of perianal fistulas with stem cells conferred a pooled healing rate of approximately 58%. The pooled healing rate after stem cell therapy was higher in Crohn’s disease-associated fistulas than in cryptoglandular fistulas and when the source of stem cells was the bone marrow compared with adipose tissues. Analysis of randomized controlled trials affirmed the efficacy of stem cells as they increased the odds of healing by approximately 80% compared with saline or placebo without increasing the likelihood of complications.

Although 43 studies were included in this systematic review, the total number of patients was approximately 1000, highlighting the small number of patients included in each study, which is expected given the experimental nature of stem cell therapy. Most studies included fistulas secondary to Crohn’s disease since perianal Crohn’s poses a special challenge owing to the poor outcomes conferred by traditional treatments [60].

The technique of stem cell therapy showed significant variations among the studies. While most studies used adipose-derived stem cells, a few studies used stromal vascular fraction or freshly collected adipose tissue. The stromal vascular fraction entails different cell types, including adipose-derived stem cells, progenitor cells, white blood cells, macrophages, and other stromal cells. Since it contains adipose-derived stem cells, the stromal vascular fraction has similar immunomodulatory and wound-healing capacities [61, 62]. While most studies used stem cells derived from the adipose tissue, six studies used bone marrow as the source of stem cells, which reflects the fact that the isolation of stem cells from the adipose tissue can be easier, safer, and affordable compared with isolation from the bone marrow [63].

The observed methodologic heterogeneity was associated with considerable statistical heterogeneity. To mitigate such heterogeneity, we performed several sensitivity analyses. Stem cell therapy conferred better healing in Crohn’s fistulas than in cryptoglandular fistulas. Stem cells maintain a local antiinflammatory environment through the secretion of some antiinflammatory molecules and, thus, stimulate the repair of damaged tissues and induce healing of fistulas [64]. Previous research confirmed that stem cells may relieve mucosal inflammation in irritable bowel disease (IBD) with mechanisms that include immunomodulation and colonization repair [65]. While this mechanism may be valid with Crohn’s fistulas, the efficacy of stem cells may be lower in cryptoglandular fistulas with inadequately drained sepsis that may hinder the action of stem cells. A previous meta-analysis [66] also reported a lower rate of healing in cryptoglandular fistulas as compared with Crohn’s fistulas (49.5% versus 53.9%). Therefore, it is advised when treating cryptoglandular fistulas with stem cell therapy to ensure adequate drainage of sepsis through drainage of any abscesses or pockets and thorough curettage of infected, unhealthy tissues and debris before the application of stem cells.

Bone marrow-derived stem cells conferred marginally better healing than adipose-derived stem cells. Although bone marrow-derived stem cells were discovered first, they were less frequently used in the treatment of anal fistulas in our review. Adipose tissues may provide a larger amount of mesenchymal stem cells, which would be easier to isolate compared with bone marrow. Despite having a similar mechanism of action, bone marrow-derived stem cells have different immunophenotypes, differentiation potential, and immunomodulatory activity from adipose-derived cells [63]. These differences may explain the better outcome of bone marrow-derived stem cells in terms of healing of anal fistulas. However, further research is needed to verify which type of stem cells would be more suitable for treating anal fistulas.

The use of stem cells was not associated with any serious adverse events. The most reported complications were anal pain and abscess, which were successfully managed in most patients with conservative measures. Furthermore, according to our meta-regression analysis, complications did not increase the risk of failure of healing. It is noteworthy that the injection of stem cells was not associated with an impairment of continence, unlike some commonly used procedures for complex anal fistulas, such as endorectal advancement flaps, that may be followed by variable degrees of continence disturbance [9]. In fact, despite the currently limited evidence, stem cell injection may contribute to an improvement in the continence state of some patients [67].

The present meta-analysis provides a summary of the pooled outcomes of stem cell therapy in anal fistulas with several sensitivity analyses that may help guide future studies and the selection of patients for stem cell treatment. However, some limitations should be acknowledged, including the small number of patients studied and the fair quality of the included studies. In addition, there was significant heterogeneity in the patient population, type, method of application of stem cells, and the definition and assessment of healing. Furthermore, there may have been variations in the outcome of stem cell therapy based on the experience of the investigators, with some of them being the developers of the technique while others are adopters.

## Conclusions

Stem cell treatment of anal fistulas was associated with promising results. Healing of anal fistulas after stem cell treatment varied according to the type of anal fistula and source of stem cells. The healing rate in Crohn’s anal fistulas was higher than in cryptoglandular fistulas. This finding suggests that the autoimmune inflammatory etiology of Crohn’s disease may respond better to autologous myoblasts than does the infectious etiology of cryptoglandular fistulas. Bone-marrow-derived stem cells were associated with marginally better outcomes than adipose-derived cells. Stem cell therapy showed a good safety profile, with anal pain and abscess being the most common adverse events after treatment.

## Supplementary Information

Below is the link to the electronic supplementary material.

Supplementary File 1 (DOCX 44 KB)

Supplementary File 2 (JPG 147 KB)

Supplementary File 3 (JPG 82 KB)

## Data Availability

Data available upon reasonable request from the first author (S.H.E.) by email at sameh200@hotmail.com.
