# Supplementary material for: Systematic review and meta-analysis of the efficacy and safety of stem cell treatment of anal fistulas
Source: Tech Coloproctol. 2025 Apr 9;29(1):100. doi: 10.1007/s10151-025-03138-y (PMC11982159; doi:10.1007/s10151-025-03138-y)
Supplement: Supplementary file 1 — (DOCX 44 KB) [file 10151_2025_3138_MOESM1_ESM.docx]

**Appendix table 1**

| **Study** | **Definition of healing** | **Type of healing** |
| --- | --- | --- |
| **Guillaumes et al, 2024** | The closing of the internal and external openings without any discharge or symptoms. | Clinical |
| **Pronk et al, 2024** | Closure of the external opening(s) and no production upon palpation and a MAGNIFICD score ≤6 on MRI. | Clinical and radiologic |
| **Swaroop et al, 2024** | Complete closure of all external openings and no collections > 2 cm on pelvic MRI at weeks 24 and 104 | Clinical and radiologic |
| **Herreros et al, 2024** | Closure of all previously draining external openings, despite gentle finger compression. MRI results with no fluid collection >2 cm in all dimensions, no edema, and no inflammation or sign of active inflammatory response; a remnant fistula tract scar could remain | Clinical and radiologic |
| **Park et al 2024** | Fistula tract closure was defined as no discharge, swelling, or pain. | Clinical |
| **Lightner et al, 2024** | Cessation in drainage and epithelialization of the external opening, and radiographic healing on magnetic resonance imaging (MRI) with an absence of a fluid collection _2 cm in any 2 of 3 dimensions | Clinical and radiologic |
| **White et al, 2024** | Absence of any clinical complaints, closure of the fistula on examination, and no secretions on palpation | Clinical |
| **Keung et al, 2023** | Absence of fistula drainage with closure of treated external openings and absence of collection (>2 cm diameter) on MRI | Clinical and radiologic |
| **Fathallah et al, 2023** | Complete closure with invisible external openings with no discharge upon pressure + fibrous sequelae with no T2 hypersignal or enhancement after the injection of gadolinium into the tract(s), and no abscess in MRI | Clinical and radiologic |
| **Wei et al, 2023** | No draining in all treated external openings despite gentle finger compression, and the absence of collections larger than 2 cm of the treated perianal fistulas in at least two of three dimensions by MRI | Clinical and radiologic |
| **Dawoud et al, 2023** | closure of all treated external openings, no secretion after external finger compression and no signs of inflammation | Clinical |
| **Arkenbosch et al, 2023** | Complete closure of all treated external opening(s) at physical examination | Clinical |
| **Dalby et al, 2023** | no external fistula opening in the perianal area was observed, no internal opening could be palpated using anorectal digital examination, and the patient had no symptoms of discharge. | Clinical |
| **Reenaers et al, 2023** | Closure of all external openings at clinical examination without discharge at digital pressure and no collection >2 cm at the pelvic MRI. | Clinical and radiologic |
| **Pak et al, 2023** | Re-epithelialization of the external fistula without inflammation, discharge, or other disease-related symptoms | Clinical |
| **Lightner et al, 2023** | Clinical and radiologic: complete cessation in drainage and the external opening was epithelialized on clinical examination + MRI with an absence of a fluid collection ≥2 cm in any 2 of 3 dimensions and a lack of edema, inflammation, or sign of active inflammatory response. | Clinical and radiologic |
| **Dozois et al, 2023** | Clinical and radiologic: absence of drainage on clinical examination when the tract and site of external opening were palpated. MRI response was defined by a decrease in the diameter and length of the T2-weighted hyperintense fistula tract on T2-weighted fast spin-echo images (expressed as percentage change from baseline), without development of abscess or additional ramifications of the treated fistula, and without increase in the Van Assche MRI perianal fistula severity score | Clinical and radiologic |
| **Furukawa et al, 2023** | Clinically confirmed closure of all treated external openings that were draining at the screening, and the absence of collections >2 cm of the treated fistulas, confirmed by a central MRI assessment. | Clinical and radiologic |
| **Guillo et al, 2022** | complete cessation of suppuration of all the external openings confirmed via magnetic resonance imaging assessment (absence of collections >2 cm in 3 axes) at weeks 12 and 48. Clinical response was defined as an evident decrease in the suppuration score | Clinical and radiologic |
| **Garcia-Olmo et al, 2022** | closure of all treated external openings that were draining at baseline despite gentle finger compression | Clinical |
| **Sørensen et al, 2022** | Clinical healing and no sign of recurrence at physical examination | Clinical |
| **Tencerova et al, 2021** | no symptom of discharge, no visible external opening in the perianal area and closed internal opening evaluated by digital rectal examination +no visible fluid conducting tract or collection at former fistula site in MRI | Clinical and radiologic |
| **Schwandner et al, 2021** | Complete healing of internal and external opening; no abscess, no symptoms | Clinical |
| **Cabalzar-Wondberg et al, 2021** | closure of all treated external openings and no drainage after gentle finger compression at the treated external opening(s) | Clinical |
| **Ascanelli et al, 2021** | External opening closed with no perianal discharge on clinical assessment | Clinical |
| **Maciel Gutiérrez et al, 2021** | Complete reepithelization of internal and external openings was achieved, accompanied by the absence of drainage or signs of inflammation | Clinical |
| **Zhang et al, 2020** | complete epithelialization of external openings (i.e., no pus outflow from the external openings under any circumstances, and no evidence of fistulas in MRI or ERUS | Clinical and radiologic |
| **Laureti et al, 2020** | closure of all treated external openings that were draining at baseline and absence of collections >3 mm of the treated perianal fistulas assessed by pelvic MRI | Clinical and radiologic |
| **Zhou et al, 2020** | Complete epithelialization of external openings (i.e., no pus outflow from the external openings under any circumstances) and no evidence of fistulas in MRI or ERUS | Clinical and radiologic |
| **Garcia-Arranz et al, 2020** | Absence of drainage through an external opening and complete re-epithelization of the external opening. | Clinical |
| **Barnhoorn et al, 2020** | Reduction in the number of draining fistulas determined by absence of discharge at physical examination and absence of collections of 2 cm directly related to the treated fistula tracts as measured by MRI | Clinical and radiologic |
| **Topal et al, 2019** | Closure of the internal and external openings without any discharge | Clinical |
| **Dige et al, 2019** | no symptoms of discharge, 2) there was no visible external fistula opening in the perineum, and 3) no internal opening could be palpated with rectal digital examination | Clinical |
| **Dozois et al, 2019** | Cessation of drainage with reepithelization of external opening | Clinical |
| **Panes et al, 2018** | closure of all treated external openings that were draining at baseline, and the absence of collections >2 cm, confirmed by blinded MRI centrally read | Clinical and radiologic |
| **Choi et al, 2017** | complete re-epithelialization of internal and external openings was achieved, accompanied by the absence of drainage or signs of inflammation. | Clinical |
| **Dietz et al, 2017** | complete cessation of drainage both spontaneously and upon gentle compression upon physical exam + decrease in the diameter and length of the T2-weighted hyperintense fistula tract on T2-weighted fast spin-echo images (percent change from baseline), without development of abscess or additional ramifcations off the treated fistula, and without increase in the Van Aasche MRI perianal fistula severity score | Clinical and radiologic |
| **Cho et al, 2015** | Complete closure of the fistula tract, including internal and external openings, without drainage or any sign of inflammation | Clinical |
| **Park et al 2015** | Decrease in fistula size of over 50% from baseline by digital rectal examination and the investigator’s satisfaction with the efficacy of the ASC treatment according to a five-point grading scale+ confirmed by MRI | Clinical and radiologic |
| **Guadalajara et al, 2012** | Clinical and radiologic healing in MRI | Clinical and radiologic |
| **de la Portilla et al, 2012** | Absence of suppuration of the fistula through the external orifice, both spontaneously and upon application of pressure, with complete reepithelization of the external orifice during clinical evaluation and absence of collections >2 cm, in three axis, directly related to the fistula tract treated, as measured by MRI | Clinical and radiologic |
| **Herreros et al, 2012** | Absence of drainage through the external openings, complete reepithelization of external openings, and the absence of collections >2 cm by MRi | Clinical and radiologic |
| **Ciccocioppo et al, 2011** | No persistence or recurrence of draining fistulas+ MRI healing | Clinical and radiologic |

**Appendix Table 2.** Results of quality assessment of non-randomized studies using ROBINS-1 tool

|  | **Bias due to confounding** | **Bias in selection of participants in the study** | **Bias in classification of intervention** | **Bias due to deviation from the intended intervention** | **Bias due to missing data** | **Bias in measurement of outcomes** | **Bias in selection of the reported result** | **Overall risk** |
| --- | --- | --- | --- | --- | --- | --- | --- | --- |
| **Guillaumes et al, 2024** | Moderate | Moderate | Low | Low | Low | Moderate | Low | High/Critical |
| **Pronk et al, 2024** | Moderate | Moderate | Low | Low | Low | Low | Low | Moderate |
| **Swaroop et al, 2024** | Moderate | Moderate | Low | Low | Low | Low | Low | Moderate |
| **Herreros et al, 2024** | Moderate | Moderate | Low | Low | Low | Low | Low | Moderate |
| **Park et al 2024** | Moderate | Moderate | Low | Low | Low | Moderate | Low | High/Critical |
| **White et al, 2024** | Moderate | Moderate | Low | Low | Low | Moderate | Moderate | High/Critical |
| **Keung et al, 2023** | Moderate | Moderate | Low | Low | Low | Low | Low | Moderate |
| **Fathallah et al, 2023** | Moderate | Moderate | Low | Low | Low | Low | Low | Moderate |
| **Wei et al, 2023** | Moderate | Moderate | Low | Low | Low | Low | Low | Moderate |
| **Dawoud et al, 2023** | Moderate | Moderate | Low | Low | Low | Moderate | Low | High/Critical |
| **Arkenbosch et al, 2023** | Moderate | Moderate | Low | Low | Low | Moderate | Low | High/Critical |
| **Dalby et al, 2023** | Moderate | Moderate | Low | Low | Low | Moderate | Low | High/Critical |
| **Reenaers et al, 2023** | Moderate | Moderate | Low | Low | Low | Low | Low | Moderate |
| **Pak et al, 2023** | Moderate | Moderate | Low | Low | Low | Moderate | Low | High/Critical |
| **Dozois et al, 2023** | Moderate | Moderate | Low | Low | Low | Low | Low | Moderate |
| **Furukawa et al, 2023** | Moderate | Moderate | Low | Low | Low | Low | Low | Moderate |
| **Guillo et al, 2022** | Moderate | Moderate | Low | Low | Low | Low | Low | Moderate |
| **Sørensen et al, 2022** | Moderate | Moderate | Low | Low | Low | Moderate | Low | High/Critical |
| **Tencerova et al, 2021** | Moderate | Moderate | Low | Low | Low | Low | Low | Moderate |
| **Schwandner et al, 2021** | Moderate | Moderate | Low | Low | Low | Moderate | Low | High/Critical |
| **Cabalzar-Wondberg et al, 2021** | Moderate | Moderate | Low | Low | Low | Moderate | Low | High/Critical |
| **Maciel Gutiérrez et al, 2021** | Moderate | Moderate | Low | Low | Low | Moderate | Low | High/Critical |
| **Zhang et al, 2020** | Moderate | Moderate | Low | Low | Low | Low | Low | Moderate |
| **Laureti et al, 2020** | Moderate | Moderate | Low | Low | Low | Low | Low | Moderate |
| **Topal et al, 2019** | Moderate | Moderate | Low | Low | Low | Moderate | Low | High/Critical |
| **Dige et al, 2019** | Moderate | Moderate | Low | Low | Low | Moderate | Low | High/Critical |
| **Dozois et al, 2019** | Moderate | Moderate | Low | Low | Low | Moderate | Low | High/Critical |
| **Choi et al, 2017** | Moderate | Moderate | Low | Low | Moderate | Moderate | Low | High/Critical |
| **Dietz et al, 2017** | Moderate | Moderate | Low | Low | Low | Low | Low | Moderate |
| **Cho et al, 2015** | Moderate | Moderate | Low | Low | Low | Moderate | Low | High/Critical |
| **Park et al 2015** | Moderate | Moderate | Low | Low | Moderate | Low | Low | High/Critical |
| **de la Portilla et al, 2012** | Moderate | Moderate | Low | Low | Low | Low | Low | Moderate |
| **Ciccocioppo et al, 2011** | Moderate | Moderate | Low | Low | Low | Low | Low | Moderate |

**Appendix Table 3.** Results of quality assessment of randomized trials using ROB-2 tool

| **Study** | **Randomization process** | **Deviation from intended intervention** | **Missing outcome data** | **Measurement of outcome** | **Selection of reported result** | **Overall risk** |
| --- | --- | --- | --- | --- | --- | --- |
| **Lightner et al, 2024** | Low | Moderate | Low | Low | Low | Moderate |
| **Lightner et al, 2023** | Low | Moderate | Low | Moderate | Low | Moderate |
| **Garcia-Olmo et al, 2022** | Low | Moderate | Moderate | Low | Low | Moderate |
| **Ascanelli et al, 2021** | Low | Moderate | Low | Low | Low | Moderate |
| **Zhou et al, 2020** | Low | Moderate | Low | Moderate | Low | Moderate |
| **Garcia-Arranz et al, 2020** | Low | Low | Low | Low | Low | Low |
| **Barnhoorn et al, 2020** | Low | Low | Low | Moderate | Moderate | Moderate |
| **Panes et al, 2018** | Low | Low | Moderate | Low | Low | Moderate |
| **Guadalajara et al, 2012** | Low | Moderate | Moderate | Moderate | Low | High |
| **Herreros et al, 2012** | Low | Low | Low | Low | Low | Low |

**Appendix table 4.** Assessment of certainty of evidence

|  | **Certainty assessment** | | | | | | | **Effect** | | **Certainty** |
| --- | --- | --- | --- | --- | --- | --- | --- | --- | --- | --- |
| **Outcome** | **№ of studies** | **Study design** | **Risk of bias** | **Inconsistency** | **Indirectness** | **Imprecision** | **Other considerations** | **Relative (95% CI)** | **Absolute (95% CI)** |  |
| Healing | 33 | observational studies | serious^a^ | very serious^b^ | not serious | not serious | none | not estimable | ------------ | ⨁◯◯◯ Very low |
| Complications | 33 | observational studies | serious^a^ | very serious^b^ | not serious | serious^c^ | none | not estimable | ------------ | ⨁◯◯◯ Very low |
| Healing | 8 | randomised trials | serious^a^ | not serious | not serious | not serious | none | **OR 1.81** (1.23 to 2.67) | **147 more per 1,000** (from 52 more to 235 more) | ⨁⨁⨁◯ Moderate |
| Complications | 8 | randomised trials | serious^a^ | not serious | not serious | not serious | none | **OR 1.00** (0.70 to 1.43) | **0 fewer per 1,000** (from 87 fewer to 80 more) | ⨁⨁⨁◯ Moderate |

**CI:** confidence interval; **OR:** odds ratio

#### Explanations

a. Most of the studies had a moderate or high risk of bias

b. The I^2^ was >75%

c. the 95% confidence interval was wide

**Appendix Table 5.** Conflict of interest disclosures in the studies

| **Study** | **Disclosures** |
| --- | --- |
| **Guillaumes et al, 2024** | The authors declare that they have no competing interests. |
| **Pronk et al, 2024** | Bemelman: Received an unrestricted grant from VIFOR, and speaker fees from Galapagos and Olympus. J. Stoker: President-elect van ESGAR. C.J. Buskens: Received an unrestricted grant from Boehringer Ingelheim and Roche, and honoraria or speaker fees from Abbvie, Tillotts, Takeda and Janssen. |
| **Swaroop et al, 2024** | The authors have no financial disclosures or conflicts of interest to declare. |
| **Herreros et al, 2024** | The treatment with darvadstrocel was financed by the Spanish National Health System. The medical writing of this article has been supported by GERM and the Autonoma University of Medicine of Madrid. |
| **Park et al 2024** | All the authors report no relevant conflicts of interest for this article. |
| **Lightner et al, 2024** | A.L.L. is a consultant for Takeda, Mesoblast, Ossium Health, Boomerang Medical; CMO of the Direct Biologics LLC. |
| **White et al, 2024** | I.W.: Takeda Pharmaceuticals consulting, training and speaker fees. Consultant for Johnson & Johnson and Medtronics. H.Y.: Research grants from Pfizer. Consultancy, advisory and speaker fees from AbbVie, Janssen-Cilag, Pfizer and Takeda. I.D.: Received advisory board and/or consulting fees and/or speaking fees from Abbott, Abbvie, Athos, Arena, Altman Research, Cambridge Healthcare, Celltrion, Celgene/BMS, Eli-Lilly, Ferring, Falk Pharma, Food Industries Organization, Gilead, Galapagos, Iterative Scopes, Integra Holdings, Janssen, Neopharm, Pfizer, Rafa laboratories, Roche/Genentech, Sangamo, Sublimity, Sandoz, Takeda, Wildbio. Grant support from Altman Research, Pfizer, BMS. N.W.: Takeda Pharmaceuticals consulting and speaker fees. Other authors—no conflicts |
| **Keung et al, 2023** | A provisional patent (AU2022901083) has been filed in Australia for use of human amnion epithelial cells in perianal fistulising Crohn’s disease with RL, CK and GM listed as co-inventors. A company (Exosome Biosciences Pty Ltd 2023) co-owned by the Hudson Institute of Medical Research, Monash University and Monash Health has been established using derivatives of human amnion epithelial cells to treat Crohn’s disease. The authors have not received any monetary payments from the company and this company had no role in any part of this study. WS, RL, CK, TCN and GM will participate in a future clinical trial affiliated with Exosome Biosciences. AG has nothing to declare |
| **Fathallah et al, 2023** | NF received research grants from Abbvie, Amgen, Tillots, Sandoz, Takeda, served as a consultant for Takeda and received consulting and speaking fees from Abbvie, Tillots and Takeda (not related to this study). LS received research grants from Takeda (not related to this study), AbbVie, Janssen, consulting and speaking fees from Takeda, AbbVie, Amgen, Janssen and Pfizer. EP received speaking fees from Takeda. MA, AD, LS and MAH have no financial or proprietary interests in any material discussed in this article. CB received research grants from Takeda (not related to this study), AbbVie and Janssen, and speaking fees from Takeda. GB received research grants from Takeda (not related to this study), AbbVie, Janssen, consulting fees from Takeda and speaking fees from AbbVie, Amgen, Janssen, Pfizer. VdP received research grants from Abbvie, Amgen, Tillots, Sandoz, Takeda, served as a consultant for Takeda and received consulting and speaking fees from Abbvie, Tillots and Takeda (not related to this study). |
| **Wei et al, 2023** | All authors declare no competing interests. |
| **Dawoud et al, 2023** | C. Dawoud received honoraria for invited presentations during satellite symposia from Takeda. M. Scharitzer received honoraria from Takeda for invited presentations during advisory board meetings and satellite symposia. A. Stift received honoraria from Takeda for invited presentations. F. Harpain reports honoraria from Takeda for invited presentations during satellite symposia. and S. Riss received honoraria fromTakeda forinvited presentations during advisory board meetings and satellite symposia. K.M. Widmann, S. Czipin and M. Pramhas declare that they have no competing interests. |
| **Arkenbosch et al, 2023** | J.H.C. Arkenbosch: no disclosures. O. van Ruler has served as invited speaker for Janssen-Cilag; and has received a research grant from Takeda, outside the submitted work. R.S. Dwarkasing: no disclosures. G. Fuhler: no disclosures. W.R. Schouten: no disclosures. M. Blussé van Oud-Alblas: no disclosures. E.J.R. de Graaf: no disclosures. A. C. de Vries has served on the advisory boards for Takeda, Janssen, Bristol Myers Squibb, Abbvie, Pfizer and Galapagos; and has received unrestricted research grants from Takeda, Janssen and Pfizer, outside the submitted work. C. J. van der Woude received grants and or fee for advisory boards and presentations from Pfizer, Abbvie, Celltrion, Falk Benelux, Takeda, Janssen and Ferring, outside the submitted work. |
| **Dalby et al, 2023** | Financial Disclosure: None reported. |
| **Reenaers et al, 2023** | The authors have no disclosures relating to the present study |
| **Pak et al, 2023** | The authors declare no conflicts of interest. |
| **Lightner et al, 2023** | Dr. Lightner is a consultant for Takeda, Mesoblast, and Ossium Health. |
| **Dozois et al, 2023** | Dr Lightner is a consultant for Takeda. Mayo Clinic and Drs Faubion, Dozois, and Dietz have a financial interest related to this research. This research has been reviewed by the Mayo Clinic Conflict of Interest Review Board and is being conducted in compliance with Mayo Clinic Conflict of Interest policies. |
| **Furukawa et al, 2023** | SF receives honoraria from Takeda Pharmaceutical and AbbVie. TM receives an honorarium from Takeda Pharmaceutical and research grants from Takeda Pharmaceutical, Kaken Pharmaceutical, Taiho Pharmaceutical, Sanofi, Chugai Pharmaceutical, Astellas Pharma, Shionogi, Mitsubishi Tanabe Pharma, Yakult Honsha, Bayer AG, Daiichi Sankyo, EA Pharma, Eli Lilly, and MSD, and the Department of Therapeutics for Inflammatory Bowel Diseases, Osaka University Graduate School of Medicine is supported by an unrestricted grant from Kinshukai Medical Corporation. RN, MS, and TY are current employees of Takeda Pharmaceutical. KW receives honoraria from AbbVie Japan, Mitsubishi Tanabe Pharma, EA Pharma, Takeda Pharmaceutical, Kyorin Pharmaceutical, Mochida Pharmaceutical, Janssen Pharmaceutical, Pfizer Japan, and Kissei Pharmaceutical. KW also receives grants including consigned/joint research expenses, scholarship donations, and course affiliations from  EA Pharma, Takeda Pharmaceutical, AbbVie Japan, Astellas Pharma, Zeria Pharmaceutical, Kyorin Pharmaceutical, Mitsubishi Tanabe Pharma, JIMRO, Otsuka Pharmaceutical, Asahi Kasei Medical, and Mochida Pharmaceutical. KF receives honoraria from Takeda Pharmaceutical, Janssen Pharmaceutical, Mitsubishi Tanabe Pharma, AbbVie, and Kyorin Pharmaceutical. KF also receives research grants from Takeda Pharmaceutical. |
| **Guillo et al, 2022** | L Guillo declares consulting fees for AbbVie. JC Grimaud has served as a consultant for AbbVie, Takeda and Janssen and as a speaker for AbbVie, Takeda, Janssen and MSD. M Serrero declares lecture and consulting fees for AbbVie, Celltrion, Ferring, Janssen, MSD, Takeda and Tillotts. JM received honorarium for educational support from FIDIA, HORIBA and MACOPHARMA. The remaining authors declare no conflict of interest. |
| **Garcia-Olmo et al, 2022** | Julian Panés has received consultant or speaker fees from Takeda Pharmaceuticals Int. Co., AbbVie, Boehringer  Ingelheim, Celgene, Celltrion, Genentech, GSK, Immunic Therapeutics, Janssen, Nestlé, Novartis, Origo Pharmaceuticals, Pandion Therapeutics, Pfizer, Progenity, Roche, Takeda Pharmaceuticals Inc., Theravance Biopharma, and Wasserman. Antonino Spinelli has received consultant fees from Takeda Pharmaceuticals Int. Co. and consultant/speaker fees from Johnson & Johnson, Janssen, and Oasis. Francesco Selvaggi has received consultant fees from Takeda Pharmaceuticals Int. Co. Dirk Lindner is an employee of Takeda Pharmaceuticals Int. Co. and has received stock/stock options. André D´Hoore has received consultant fees from Takeda Pharmaceuticals Int. Co. and Johnson & Johnson. Matthias Binek is an employee of Takeda Pharmaceuticals Int. Co. and has received stock/stock options. Inmaculada Gilaberte is an employee of Takeda Pharmaceuticals Int. Co. and has received stock/stock options. Damián Garcia-Olmo has received consultant fees from Takeda Pharmaceuticals Int. Co. and is a named inventor on patents related to this study. |
| **Sørensen et al, 2022** | S.P.S. is owner and CEO of Blue Cell Therapeutics. The other authors declare that they have no conflicts of interest. |
| **Tencerova et al, 2021** | The authors have nothing to disclose. |
| **Schwandner et al, 2021** | The author had honoraria from Takeda and Medtronic for invited presentations during advisory board meetings and satellite symposia. Neither funding nor financial support was administered for the current study |
| **Cabalzar-Wondberg et al, 2021** | PS: travel support from Falk, UCB and Pfizer and advisory board honorarium from Pfizer, Takeda and Janssen-Cilag. GR: consultancy to Abbvie, Augurix, BMS, Boehringer, Calypso, Celgene, FALK, Ferring, Fisher, Genentech, Gilead, Janssen, MSD, Novartis, Pfizer, Phadia, Roche, UCB, Takeda, Tillots, Vifor, Vital Solutions and Zeller; speaker's honoraria from Astra Zeneca, Abbvie, FALK, Janssen, MSD, Pfizer, Phadia, Takeda, Tillots, UCB, Vifor and Zeller; educational grants and research grants from Abbvie, Ardeypharm, Augurix, Calypso, FALK, Flamentera, MSD, Novartis, Pfizer, Roche, Takeda, Tillots, UCB and Zeller. LB: fees for consulting/advisory board from Abbvie, MSD, Vifor, Falk, Esocap, Calypso, Ferring, Pfizer, Shire, Takeda, Janssen, Ewopharma. DC-W: speaker's honoraria and advisory board honorarium from Takeda. MT: fees for consulting/advisory board from Takeda; speaker’s honoraria from Intuitive Surgical. |
| **Ascanelli et al, 2021** | Financial Disclosures: None reported. |
| **Maciel Gutiérrez et al, 2021** | Top-Health provided the allogeneic mesenchymal stem cells for the study. The cost of the hospital stay and surgery were covered by the patient’s health insurance. |
| **Zhang et al, 2020** | The authors declare that they have no competing interests. |
| **Laureti et al, 2020** | Not reported |
| **Zhou et al, 2020** | The authors declare that they have no competing interests. |
| **Garcia-Arranz et al, 2020** | D.G.-O. is a member of the Advisory Board of Tigenix S. A. U. and has received fees from Takeda. D.G.-O. and M.G.-A. have applied for two patents related to this study entitled “Identification and isolation of multipotent cells from nonosteochondral mesenchymal tissue” (WO 2006/057649) and “Use of adipose tissue-derived stromal stem cells in treating fistula” (WO 2006/136244). D.G.-O., M.G.-A., and H.G. are shareholders of Biosurgery, an educational company providing services to Takeda. The other authors indicated no potential conflicts of interest. |
| **Barnhoorn et al, 2020** | MCB, MNJMW, HF, PWJM, IM, BAB, LEMO, GD, DLR, JZZ, HWV, WEF, DWH, KCMJP declare no conflict of interest. CJW received grants from Pfizer, Takeda, and Tramedico; and served on advisory boards from Takeda, Abbvie, and Janssen. AEMJ received a grant from Takeda; and received speaker fee from Janssen, Cilag, and Takeda |
| **Topal et al, 2019** | Not reported |
| **Dige et al, 2019** | The authors disclose no conflicts. |
| **Dozois et al, 2019** | Dr Faubion is a Consultant and on the Advisory Board for AbbVie, a consultant for Boehringer Ingelheim Pharma and Celgene, Consultant and Advisory Board member for Janssen, and a consultant for Robarts, Takeda, and MediBeacon. Dr Lightner is a consultant for Takeda. Dr Dietz is an inventor of technology used as a tool in this research; the technology has been licensed to a commercial entity (PLTMax; Mill Creek LifeScienes). Dr Dietz and the Mayo Clinic have equity in the company and have contractual rights to receive royalties from the licensing of this technology. Greg W. Butler is an inventor of technology used as a tool in this research; the technology has been licensed to a commercial entity (PLTMax; Mill Creek LifeScienes). Greg Butler and the Mayo Clinic have equity in the company and have contractual rights to receive royalties from the licensing of this technology. Joel G. Fletcher is a consultant for Medtronics and received a grant to institution from Siemens Healthineers. |
| **Panes et al, 2018** | The authors disclose the following: Julián Panés has received personal fees from TiGenix, AbbVie, Boehringer Ingelheim, Celgene, Galapagos, Genentech-Roche, GSK, Janssen, MSD, Novartis, Oppilan, Pfizer, Takeda, Theravance, and Vivelix. Damián García-Olmo has received personal fees from TiGenix, and has a patent and a patent pending. Gert Van Assche has received personal fees from TiGenix and grants and personal fees from AbbVie, Takeda, MSD, Janssen, Pfizer, Ferring, and Genentech-Roche. Jean Frederic Colombel reports grants from AbbVie, Janssen and Janssen, and Takeda; other fees from Boehringer Ingelheim, Celgene Corporation, Celtrion, Enterome, Ferring, Genentech, Medimmune, Merck & Co., Pfizer, Protagonist, Second Genome, Seres, Shire, Theradiag, Amgen, Intestinal Biotech Development, and Genefit. Walter Reinisch has received personal fees as a speaker from Abbott Laboratories, AbbVie, Aesca, Aptalis, Astellas, Centocor, Celltrion, Danone Austria, Elan, Falk Pharma GmbH, Ferring, Immundiagnostik, Mitsubishi Tanabe Pharma Corporation, MSD, Otsuka, PDL, Pharmacosmos, PLS Education, Schering-Plough, Shire, Takeda, Therakos, Vifor, and Yakult; personal fees as a consultant from Abbott Laboratories, AbbVie, Aesca, Amgen, AM Pharma, Astellas, Astra Zeneca, Avaxia, Roland Berger GmbH, Bioclinica, Biogen IDEC, Boehringer Ingelheim, Bristol-Myers Squibb, Cellerix, Chemocentryx, Celgene, Centocor, Celltrion, Covance, Danone Austria, Elan, Ernest & Young, Falk Pharma GmbH, Ferring, Galapagos, Genentech, Gilead, Grünenthal, ICON, Index Pharma, Inova, Janssen, Johnson & Johnson, Kyowa Hakko Kirin Pharma, Lipid Therapeutics, Mallinckrodt, MedImmune, Millennium, Mitsubishi Tanabe Pharma Corporation, MSD, Nestle, Novartis, Ocera, Otsuka, Parexel, PDL, Pharmacosmos, Pfizer, Procter & Gamble, Prometheus, Robarts Clinical Trial, Sandoz, Schering-Plough, Second Genome, Setpointmedical, Sigmoid, Takeda, Therakos, TiGenix, UCB, Vifor, Zealand, Zyngenia, and 4SC; personal fees as an advisor from Abbott Laboratories, AbbVie, Aesca, Amgen, AM Pharma, Astellas, Astra Zeneca, Avaxia, Biogen IDEC, Boehringer Ingelheim, Bristol-Myers Squibb, Cellerix, Chemocentryx, Celgene, Centocor, Celltrion, Danone Austria, Elan, Ferring, Galapagos, Genentech, Grünenthal, Inova, Janssen, Johnson & Johnson, Kyowa Hakko Kirin Pharma, Lipid Therapeutics, MedImmune, Millennium, Mitsubishi Tanabe Pharma Corporation, MSD, Nestle, Novartis, Ocera, Otsuka, PDL, Pharmacosmos, Pfizer, Procter & Gamble, Prometheus, Sandoz, ScheringPlough, Second Genome, Setpointmedical, Takeda, Therakos, TiGenix, UCB, Zealand, Zyngenia, and 4SC; and grants from Abbott Laboratories, AbbVie, Aesca, Centocor, Falk Pharma GmbH, Immundiagnostik, and MSD. Daniel C. Baumgart reports grants, personal fees, and nonfinancial support from Shire, AbbVie, MSD, Takeda, Biogen, Dr. Falk, Ferring, Recordati, GenentechRoche, Janssen, Pfizer, BMS; personal fees and nonfinancial support from Foreward Pharma and TiGenix; and grants from Nestle, Hitachi, Shield Therapeutics, and Celgene. Axel Dignass reports consultancy fees from AbbVie, MSD, Ferring, Genentech-Roche, Takeda, Pharmacosmos, Vifor, Falk, Mundipharma, Janssen, Allergosan, Hospira, Robarts, Pfizer, Sandoz/ Hexal, and Celgene; grants from Institut für Gemeinwohl and Stiftung Leben mit Krebs; payment for lectures from Falk Foundation, Ferring, MSD, AbbVie, Otsuka, Vifor, Immunodiagnostik, Jansen-Cilag, Med Update GmbH, Medice, Pfizer, Mundipharma, Tillotts, and Hospira; manuscript preparation fees from Falk Foundation, Wiley, Thieme, Allergosan, and Takeda; and educational presentation development fees from Pharmacosmos, Falk Foundation, and Tillotts. Maria Nachury has received personal fees from TiGenix and Boehringer Ingelheim; and personal fees and nonfinancial support from AbbVie, MSD, and Takeda. Marc Ferrante has received nonfinancial support from TiGenix; grants, personal fees, and nonfinancial support from Takeda;and personal fees and nonfinancial support from MSD, Janssen, AbbVie, Chiesi, Tillotts, Ferring, Falk, Mitsubishi, Zeria, and Boehringer Ingelheim. Lili Kazemi-Shirazi reports nonfinancial and other support from TiGenix study; other support from SigmaTau and Sanofi; personal fees from MSD, AbbVie, Ferring, MerckSerono/Dr Falk, Chiesi, Novartis, Roche, Abbott, Phadia Austria/Thermo Fisher Scientific, and CSL-Behring; and nonfinancial support from Mylan, Abbott, MSD, Gilead, MerckSerono/Dr Falk, and Novartis. Marie Paule Richard, Mary Carmen Diez, Ignacio Tagarro, and Anne Leselbaum have received personal fees from TiGenix. Silvio Danese has received personal fees from AbbVie, Allergan, Sandoz, UCB, Boehringer Ingelheim. Vifor, Celltrion, Sandoz, MSD, Takeda, Janssen, Mundipharma, Hospira, Ferring, Merck, and Pfizer. Jean C. Grimaud, Fernando de la Portilla, and Eran Goldin declare no competing interests |
| **Choi et al, 2017** | The authors declare that they have no conflict of interest. |
| **Dietz et al, 2017** | The authors declared the following potential conflicts of interest with respect to the research, authorship, and/or publication of this article: Dr. Allan Dietz and Greg Butler are inventors of technology used as a tool in this research; the technology has been licensed to a commercial entity (PLTMax; Mill Creek LifeScienes). ABD and Mayo Clinic have equity in the company and ABD and GWB have contractual rights to receive royalties from the licensing of this technology. ABD has governance responsibilities within this company. These conflicts have been disclosed to and are managed by the Mayo Clinic Conflict of Interest Board and are included here as directed by them. No other authors have a conflict to report. |
| **Cho et al, 2015** | M.H.K. and H.Y.J. are compensated employees of Anterogen Co. Ltd. The other authors indicated no potential conflicts of interest. |
| **Park et al 2015** | Not reported |
| **Guadalajara et al, 2012** | This clinical trial has been sponsored by Cellerix S.L. Damian García-Olmo is a holder of the UAM–Cellerix Chair of Cell Therapy and Regenerative Medicine to which Cellerix contributes 40,000€ per year. UAM and Cellerix S.A. share patent rights to Cx401. García-Olmo is a member of the advisory board of Cellerix S.A. M. Garcia-Arranz and D. García-Olmo are inventors in two patents related to Cx401 entitled “Identification and isolation of multipotent cells from non-osteochondral mesenchymal tissue” (10157355957US) and “Use of adipose tissue-derived stromal stem cells in treating fistula” (US11/167061) |
| **de la Portilla et al, 2012** | There are no competing interests |
| **Herreros et al, 2012** | Prof D. García-Olmo is Chairman of the UAM Cellerix Cell Therapy and Regenerative Medicine Department, to which Cellerix has contributed €40,000 per year. UAM and Cellerix S.A. share patent rights to Cx401 (Adipose Derived Stem Cells). Prof D. García Olmo is a member of the advisory Board of Cellerix. M. Garcia-Arranz and Prof D. García-Olmo have applied for 2 patents related to Cx401 titled “Identification and isolation of multipotent cells from non-osteochondral mesenchymal tissue” (WO 2006/057649) and “Use of adipose tissue-derived stromal stem cells in treating fistula” (WO 2006/136244). |
| **Ciccocioppo et al, 2011** | Competing interests None. |
