# Supplementary figures and images for: Systematic review and meta-analysis of the efficacy and safety of stem cell treatment of anal fistulas
Source: Tech Coloproctol. 2025 Apr 9;29(1):100. doi: 10.1007/s10151-025-03138-y (PMC11982159; doi:10.1007/s10151-025-03138-y)

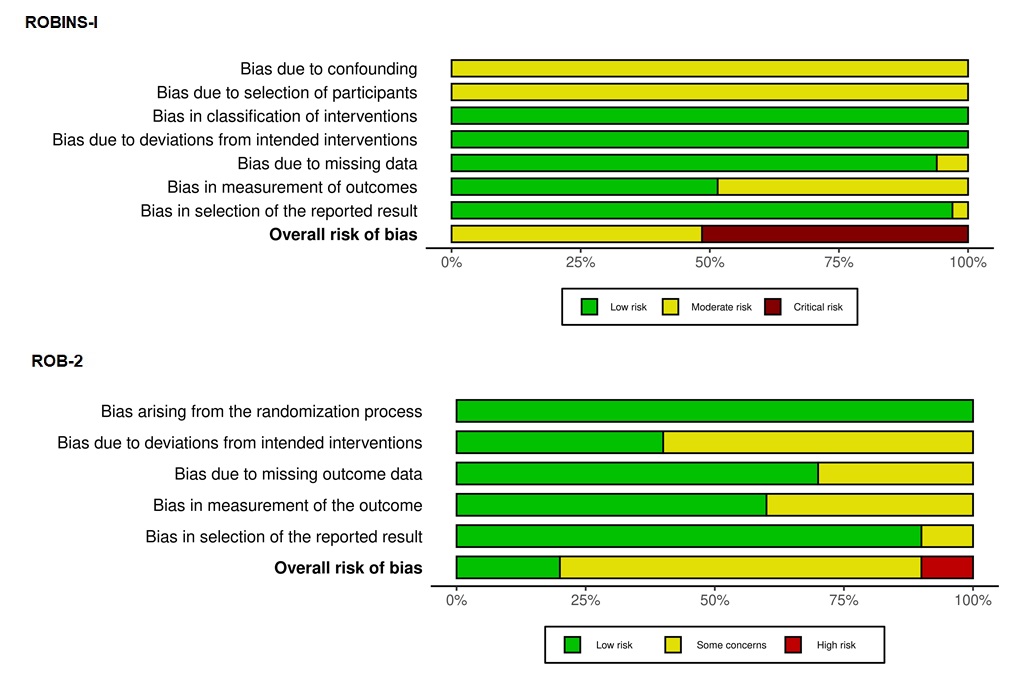

Supplement: Supplementary file 2 — (JPG 147 KB) [file 10151_2025_3138_MOESM2_ESM.jpg]

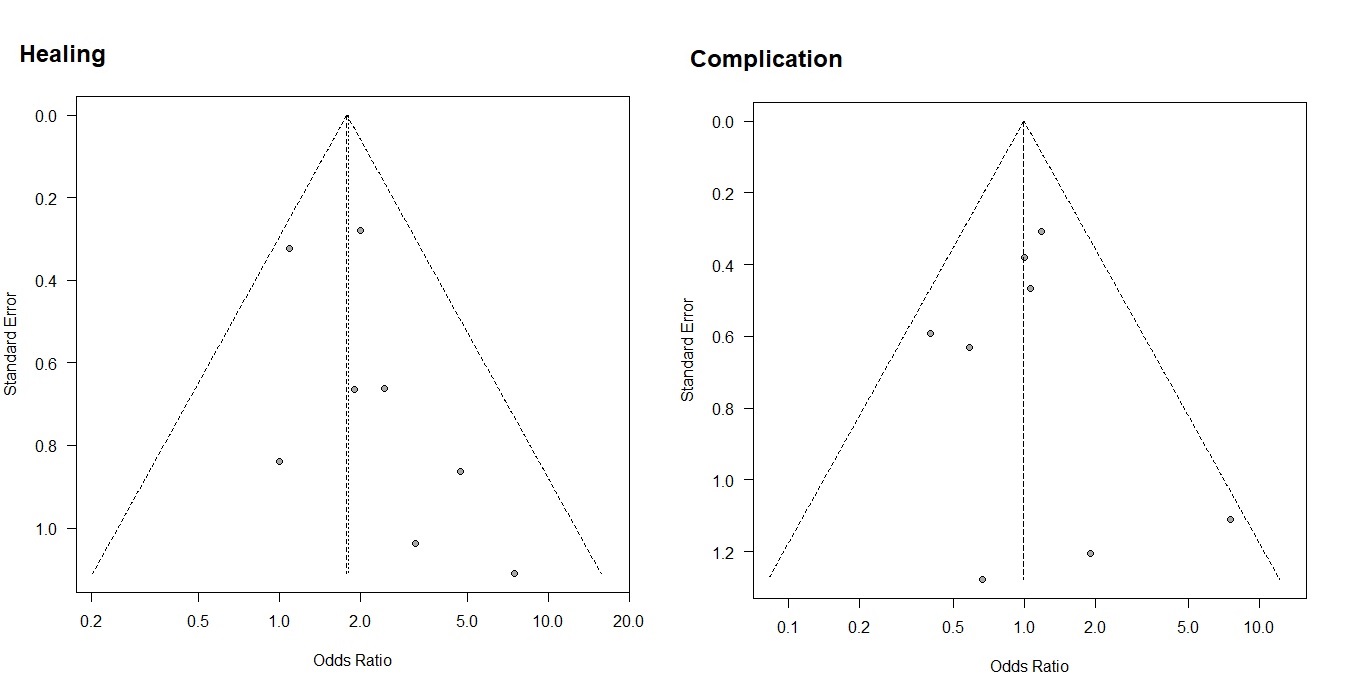

Supplement: Supplementary file 3 — (JPG 82 KB) [file 10151_2025_3138_MOESM3_ESM.jpg]
